# Rigor and standardization of extracellular vesicle research: Paving the road towards robustness

**DOI:** 10.1002/jev2.12037

**Published:** 2020-12-14

**Authors:** Rienk Nieuwland, Juan M. Falcón‐Pérez, Clotilde Théry, Kenneth W. Witwer

**Affiliations:** ^1^ Laboratory of Experimental Clinical Chemistry, Department of Clinical Chemistry, Amsterdam UMC, Location AMC University of Amsterdam Amsterdam The Netherlands; ^2^ Vesicle Observation Centre, Amsterdam UMC, Location AMC University of Amsterdam Amsterdam The Netherlands; ^3^ Center for Cooperative Research in Biosciences (CIC bioGUNE) Basque Research and Technology Alliance (BRTA) Exosomes Laboratory Derio Spain; ^4^ Centro de Investigación Biomédica en Red de enfermedades hepáticas y digestivas (CIBERehd) Madrid Spain; ^5^ IKERBASQUE Basque Foundation for Science Bilbao Spain; ^6^ Institut Curie INSERM U932 PSL Research University Paris France; ^7^ Department of Molecular and Comparative Pathobiology Johns Hopkins University School of Medicine Baltimore Maryland USA; ^8^ Department of Neurology Johns Hopkins University School of Medicine Baltimore Maryland USA

Accompanying the growing awareness that extracellular vesicles (EVs) are useful for diagnostics and therapeutics is the realization that EV applications must be established with rigor, reproducibility, and standardization. The small size and heterogeneity of most EVs are well‐known barriers to rigorous studies. However, progress is also hampered by the largely unknown influence of a host of pre‐analytical variables and a lack of quality controls, differences in EV separation and characterization techniques (van Deun et al., 2017), a general lack of dedicated reference materials and standards (Welsh et al., 2020), and analyses that are poorly defined and thus irreproducible. The goal of this Editorial is to summarize the past, present, and future contributions of the International Society for Extracellular Vesicles (ISEV) to improving rigor and standardization in EV research. We highlight in particular the Rigor and Standardization Subcommittee and the recently published results of a survey on the methods used for separation and characterization of EVs (Royo, Théry, Falcón‐Pérez, Nieuwland, & Witwer, 2020). Major challenges remain. Even so, we predict that within a few years, the expansion of an ISEV infrastructure dedicated to rigor and standardization will enable us to turn the ugly duckling of current challenges into a beautiful swan of comparable and reproducible EV measurement results. Because we are working in a new and developing field of research, ISEV has the unique opportunity to set high standards for rigor, and we may even lead the way for other research fields and societies.

Rigor and standardization with community input have been part of the ISEV mission since its founding. Impetus for founding a society arose with a 2011 meeting in Paris that was organized by Clotilde Théry and Graça Raposo. The incipient community voted to emphasize 'extracellular vesicles' in the name of the society rather than limiting focus to specific subtypes of EVs. Following the first annual meeting of ISEV in 2012, the first ISEV Workshop was organized and held in October, 2012 in New York City. This Workshop gave rise to the first two ISEV position papers, on standardization of sample collection and processing (Witwer et al., 2013) and RNA analysis (Hill et al., 2013). Subsequent workshops and co‐sponsored events have given birth to additional position papers and other products, most of them published in the Journal of Extracellular Vesicles (JEV) (Lötvall et al., 2012; Théry, Gho, & Quesenberry, 2019), that provide guidance on topics ranging from non‐mammalian EVs (Soares et al., 2017) to EV RNA (Mateescu et al., 2017) and membranes (Russell et al., 2019) to biomarker considerations (Clayton et al., 2018) and therapeutic uses of EVs (Lener et al., 2015; Reiner et al., 2017). In 2014, the first position statement was published on the '*Minimal experimental requirements for definition of extracellular vesicles*' (MISEV) (Lötvall et al., 26913). Following a community guidance survey (Witwer et al., 2017) and a series of intensive information‐gathering surveys and exchanges with the ISEV board, MISEV was updated as the 382‐author 'MISEV2018' (Théry et al., 2018). The goal of these manuscripts has not changed over the years, because it was and still is a challenge to purify and detect EVs, and to attribute specific biological cargo and functions to EVs.

Working towards rigor and standardization, ISEV is not alone. Over the years, formal and informal collaborations with other societies and groups have coalesced. An early development was the founding of the Extracellular Vesicle Flow Cytometry Working Group (www.evflowcytometry.org), which first met at the ISEV2015 annual meeting. Members of ISEV, ISTH (International Society on Thrombosis and Haemostasis) and ISAC (International Society for Advancement of Cytometry) have prepared guidelines to improve the reproducibility of EV detection by flow cytometry, culminating in the 2020 position paper '*MiFlowCyt‐EV: a framework for standardized reporting of extracellular vesicles flow cytometry experiments*' (Welsh et al., 2020). Additional papers are planned, including an educational manuscript about 'do's and don't's' of EV flow cytometry. A guidelines publication was produced by several ISEV Board of Directors (BoD) members with members of ISTH and the American Heart Association (AHA) (Coumans et al., 2017). ISEV has worked with groups such as SOCRATES and the International Society for Cell and Gene Therapy (ISCT) on issues of clinical translation of EVs (Börger et al., 2020; Witwer et al., 2019). Importantly, the EV‐TRACK consortium and knowledgebase, led by the Hendrix group, was launched to improve transparent reporting of EV research and is endorsed by ISEV/JEV (van Deun et al., 2017).

Amidst these positive developments, a perceived need for a more formal infrastructure to promote rigor in EV science led to formation of a dedicated subcommittee of the ISEV BoD in 2019. At the latest during the ISEV Workshop on EVs as Biomarkers (Birmingham, UK, 2017) and an associated strategic planning session of the BoD, it became clear to ISEV members that specific challenges in the field could be best addressed by a supervised combination of crowd sourcing and task forces, much as was implemented for MISEV2018. Planning continued through the ISEV Workshop on Theranostics (Guangzhou, China, 2018), and the Rigor and Standardization Subcommittee was formally announced and first met during ISEV2019 (Kyoto, Japan). In this subcommittee, Co‐Chairs Juan Manuel Falcon‐Perez and Rienk Nieuwland lead a team consisting of several core members and the leaders of topic‐specific task forces.

How does the Rigor and Standardization Subcommittee work with task forces? Several task forces came into being along with the subcommittee. However, community members can also propose task forces through an online application system (see Text Box 1). Proposals are reviewed by the subcommittee. Task forces are not meant to be permanent bodies, but rather ad hoc groups devoted to specific questions. Since the work of the task forces must be focused, well‐planned, and guided by experts in EV studies but also in related fields, not all proposals are immediately selected, and actual formation can take time. Task forces may examine specific research topics including sources of EVs (biofluids, tissues, cell culture), but they may also deal with regulatory issues, such as the ISEV Regulatory Affairs task force. ISEV supports task forces in several ways, per Text Box 1. Examples of current task forces can be found at: https://www.isev.org/page/RigorStandardization.

Task forces are expected to produce products, the nature of which is determined by the scope and needs of the questions they address, ranging from ISEV position papers (which require endorsement by the ISEV BoD) to primary research output (e.g., in the case of interlaboratory comparisons), methodologic protocols, literature reviews, online educational materials, and more. Thus far, products have included a 'roadmap' for collection, handling and storage of blood EVs (Clayton et al., 2019), a workshop on EV standardization and reference materials (Ghent, Belgium, 2019), and a manuscript about reference materials for EV research (Welsh et al., 2020). The contribution of metrologists to this latter manuscript illustrates that traceability of measurement results is coming into reach. In parallel, dedicated reference materials for EV research are being developed, such as engineered or recombinant EVs (Geeurickx et al., 2019; Görgens et al., 2019) and synthetic nanoparticles (van der Pol, Coumans, Sturk, Nieuwland, & van Leeuwen, 2014).

Another goal of the Rigor and Standardization Subcommittee is to monitor method and technology development and adoption. In 2015, ISEV members were surveyed on methods used for isolation and characterization of EVs, and the results were published in 2016 (Gardiner et al., 2016). A second 'pulse‐taking' of the community was implemented in 2019 and published by Rigor and Standardization (Royo et al., 2020). Although neither the questions nor the respondents were identical, and thus a perfect comparison cannot be made for most questions and answers, the results of the two surveys can nevertheless provide some insights into trends in EV research methodologies and goals. In 2019, more than 600 full or partial responses were recorded, and approximately twice as many full responses were received compared with 2015. The new survey reveals that an increasing number of EV researchers are using new and more advanced separation and characterization methods, have started measuring quality control parameters, and are applying more EV characterization methods in parallel. Thus, there is clearly a growing awareness amongst ISEV members that 'rigor and standardization' are essential and a prerequisite to improve the credibility of our research.

**EV source**. A significantly larger proportion of respondents in 2019 versus 2015 reported using serum‐free cell culture media, blood plasma, blood serum, and urine as sources of EVs. The 2019 survey also included a 'tissue' source, and more than 20% of respondents reported using tissue to obtain EVs.

**EV separation methods**. While differential ultracentrifugation remains the workhorse of the field, its use appears to have declined slightly (Gardiner et al., 2016, Royo et al., 2020). In contrast, use of size exclusion chromatography has more than doubled, and gradient ultracentrifugation and affinity methods have also increased (Gardiner et al., 2016, Royo et al., 2020). Polymer‐based precipitation is used by more than 20% of respondents, although it is perhaps the 'dirtiest' method available unless combined with other approaches. In 2019, the survey also provided options for microfluidics, field‐flow, tangential flow for the first time (Royo et al., 2020).

**Biobanking and rigor of quality control**. The recent survey queried archiving in biobanks and use of biobanked samples, along with implementation of sample quality control measures. Interestingly, the results suggest that researchers who contribute materials to biobanks give more attention to quality control. A majority of these 'biobankers' indicated that they performed quality control of their source material prior to EV separation and that they also assessed recovery of EVs after separation, while only a minority of 'non‐biobankers' performed these assays.

**EV biomolecules of interest**. The 2015 survey asked about 'downstream applications,' while the 2019 survey focused on molecules of interest (Gardiner et al., 2016, Royo et al., 2020). Nevertheless, we can make some comparisons. It appears that while RNA assays were the most popular reported downstream assay in 2015, in 2019, proteins appear to predominate slightly. This is not due to a decline in interest in RNA, though, as RNA garners even more interest than in 2015. Also, lipids/lipidomics were of interest to 5% of respondents in 2015, but to almost 25% in 2019. Thus, we are seeing an increase in measurements of various EV constituents across the board, and we view this positively. It is not as if any particular molecular category has 'lost out;' rather, there has been an awakening of study of multiple analytes. Broader cataloguing of EV molecular composition will likely provide new insights into mechanisms of EV biogenesis, identify previously overlooked biomarkers, and unravel more about the functions of EVs.

**EV characterization methods**. Major techniques for EV characterization, such as Western blotting, single particle tracking, electron microscopy, and flow cytometry, appeared to maintain their relative ranks, roughly, from 2015 to 2019 (Gardiner et al., 2016, Royo et al., 2020). Importantly, though, respondents in 2019 reported using more–and more diverse–characterization methods. Most 2019 researchers used four to six characterization methods, whereas most 2015 respondents used three or fewer. We suspect that the increase in the number of characterization methods has been driven at least in part by the MISEV efforts. However, an increasing number of dedicated EV characterization methods have also become more widely reported and commercially available.

**EV normalization**. A new subject in the 2019 survey was normalization: how to determine how much material to use in functional assays. Here, participants indicated use of EV number and protein concentration primarily, followed by number of cells and volume of source material from which EVs were harvested. Spike‐ins and housekeeping markers were used by relatively few respondents. The survey also found that reported in vivo functional assay use increased by more than 25% compared with 2015 levels (Gardiner et al., 2016; Royo et al., 2020). It cannot be concluded from the survey answers, though, which in vivo models are involved, nor can we know if more widespread use of such assays means an overall increase in the total number of subjects employed.

How about the future? Where will we be in a few years? What should be expected from a hypothetical third ISEV survey on separation and characterization? As outlined at the beginning, apart from the fact that EVs are small and heterogeneous, major obstacles are (i) biospecimen variability, (ii) pre‐analytical variables, (iii) poor analytics and (iv) lack of reference materials and standards. Size and heterogeneity of EVs are constant variables that will always be with us, and the same holds true for variation between biospecimens. Pre‐analytical variables (more than 40 pre‐analytical variables for blood collection and handling include, for example anti‐coagulation and plasma centrifugation conditions), are difficult to approach, but at least pre‐analytical variables are now recorded in EV‐TRACK. To what extent these variables affect EV outcomes is not yet entirely known because analytics are not yet in place. The situation is changing rapidly, however, and we expect that substantial additional progress will be made by the next survey. The course of progress can perhaps best be illustrated by developments in EV detection by flow cytometry.

At present, dedicated reference materials for flow cytometry detection of EVs are being developed together with industry and metrologists (www.metves.eu), allowing full calibration of instruments. Calibration is a prerequisite for comparing measurement results. Furthermore, software has been developed, based on physical models, to correct for differences in the sensitivity (i.e. optical configuration) of different instruments. The latter have been tested already in a worldwide inter‐laboratory comparison study (van der Pol et al., 2018), a new study is on its way, and robust data reporting is now facilitated by MiFlowCyt‐EV (Welsh et al., 2020). Due to this progress, producers of flow cytometers have also become interested, and several new types of flow cytometers have and are being developed with improved sensitivity for EV detection. Thus, we hope that within 1 or 2 years, we will have at least one piece of analytics in place that allows reliable quantification of the concentration of cell‐type specific EVs in biofluids directly, that is, without isolation. Once analytics are in place, we can study the effects of pre‐analytics, such as handling and storage conditions, but also of separation methods. Furthermore, by assessing the quality of the prepared (EV‐containing) biofluids into account, for example, by measuring contaminants, more robust and reliable results can be obtained. Thus, we expect that in the third ISEV survey, we will have set at least one but perhaps more examples of robust and reproducible EV research.

In conclusion, since 2015, tremendous progress has been made regarding EV separation and characterization methods. There is a growing awareness amongst EV researchers about the relevance of 'rigor and standardization’, and in parallel an infrastructure has been developed that likely will place EV research within the front row of scientific fields that produce truly reliable and reproducible data. We will finally be able to answer long‐standing biological questions in a rigorous fashion. For example, reliable measurements of EVs concentrations are needed to resolve the perennial debate about how many microRNAs are contained in EVs and whether these have a function (Albanese et al., 2020; Chevillet et al., 2014, Pegtel et al., 2010; Valadi et al., 2007). Reliable and reproducible data are also a prerequisite for clinical multicentre studies, thus paving the road to diagnostics and therapeutics. To make a success of rigor and standardization, active participation of EV researchers is essential, as are collaborations with other societies and agencies. We strongly and enthusiastically encourage the ISEV community to participate in this process.

**Text Box 1**

**ISEV Rigor and Standardization task force proposals are invited through the link** https://www.isev.org/page/TaskForceProposals

Submitters are asked to provide the following:

‐ Topic and scope

‐ Chair(s): (usually one, max two); Chair becomes a member of the Rigor and Standardization committee

‐ Contributing members (max 30) with evidence of expertise and keeping geographic/subject/gender diversity in mind; non‐ISEV members are encouraged with justification

‐ Timeline (1‐2 years): might be renewed but finite (not permanent bodies)

‐ Anticipated products

Applications will be reviewed at least twice a year by the R&S subcommittee. Not all will be approved. Committee or Board may make changes or suggestions.

**What ISEV provides to task forces**:

‐ Coordination of and between task forces, including a yearly meeting at the annual ISEV conference and up to three teleconferences per year

‐ Communication tools (teleconferences, web page)

‐ Potential endorsement and/or financial support of published products

‐ Guidance for engagement with other standardization committees, agencies, societies

‐ Visibility and transparency

‐ Possibility of organizing workshops/seminars/hands‐on training sessions
